# Development and validation of a ferroptosis-related lncRNAs signature to predict prognosis and microenvironment for melanoma

**DOI:** 10.1007/s12672-022-00581-3

**Published:** 2022-11-12

**Authors:** Shuai Ping, Ruining Gong, Ke Lei, Gong Qing, Guangheng Zhang, Jianghai Chen

**Affiliations:** 1 Department of Gastroenterology, Tumor Immunology and Cytotherapy, Medical Research Center, The Affiliated Hospital of Qingdao University, No. 1677 Wutaishan Road, Huangdao District, Qingdao, 266000 China; 2 Tumor Immunology and Cytotherapy, Medical Research Center, The Affiliated Hospital of Qingdao University, No. 1677 Wutaishan Road, Huangdao District, Qingdao, 266000 China; 3 Department of Orthopaedics, Liyuan Hospital, Tongji Medical College, Huazhong University of Science and Technology, Wuhan, 430077 China; 4 Department of Hand Surgery, Union Hospital, Tongji Medical College, Huazhong University of Science and Technology, Wuhan, 430022 Hubei China

**Keywords:** Ferroptosis, lncRNA, Melanoma, Prognosis, Tumor microenvironment

## Abstract

Ferroptosis plays an important role in cancer. However, studies about ferroptosis-related lncRNAs (FRLs) in skin cutaneous melanoma (SKCM) are scarce. Moreover, the relationship between prognostic FRLs and tumor microenvironment (TME) in melanoma remains unclear. This study investigates the potential prognostic value of FRLs and their association with TME in SKCM. The RNA-sequencing data of SKCM were downloaded from The Cancer Genome Atlas (TCGA) database. Melanoma patients were randomly divided into training and testing groups in a 1:1 ratio. A signature composed of 19 FRLs was developed by the least absolute shrinkage and selection operator (LASSO) regression analysis to divide patients into a low-risk group with a better prognosis and a high-risk group with a poor prognosis. Multivariate Cox regression analysis suggested that the risk score was an independent prognostic factor. The Area Under Curve (AUC) value of the risk score reached 0.768 in the training group and 0.770 in the testing group. Subsequent analysis proved that immune-related signaling pathways were significantly enriched in the low-risk group. The tumor immune cell infiltration analysis demonstrated that melanoma in the high-risk group tended to be immunologically “cold”. We identified a novel FRLs signature which could accurately predict the prognosis of patients with melanoma.

## Introduction

Melanoma, a malignant tumor derived from melanocytes, is highly aggressive and characterized by a strong tendency for invasiveness and metastasis [1]. Although rare, melanoma has a much higher mortality rate than other common skin cancers [2]. SKCM is primarily caused by excessive ultraviolet radiation exposure, severe sunburns, and external stimulation [3]. Nowadays, various methods are used in the management of melanoma, such as surgery and immunotherapy [4–6]. Surgery remains the primary approach for the treatment of melanoma with a high cure rate; however, the mortality rate of advanced melanoma remains high [7]. The application of immune checkpoint inhibitors (ICIs) has improved melanoma patients’ survival significantly. However, malignant melanoma’s prognosis continues to be poor due to the disease's resistance to chemotherapy, radiotherapy, immunotherapy, and the occurrence of distant metastasis [8]. Hence, there is a need for novel diagnostic biomarkers for predicting the prognosis of and treatment effects of melanoma.

Ferroptosis is a novel form of programmed cell death that is caused by iron-dependent lipid peroxidation [9, 10]. Behrouz Hassannia et al. find that ferroptosis is associated with tumor occurrence and progression and that inducing ferroptosis is a new strategy for treating tumors, especially drug-resistant tumors [11]. The ferroptosis resistance decreases the therapeutic efficacy of sorafenib, leading to a poor prognosis for patients with hepatocellular carcinoma [12, 13]. It has been proved that suppression of ferroptosis contributed to poor outcomes in colorectal cancer [14]. Similarly, ferroptosis plays a significant role in the management of melanoma. Recent Yongfei Yang et al. have revealed that the depletion of NEDD4 could promote the erastin-induced ferroptosis of melanoma cells [15].

Additionally, another research has proved that miR-137 enhanced the therapeutic efficacy via increasing melanoma ferroptosis [16]. These results imply that inducing ferroptosis may be a therapeutic method in the treatment of melanoma. However, only a few studies explored the correlation between melanoma and ferroptosis [17, 18]. Hence, it is important to identify new FRLs biomarkers for predicting the prognosis of patients with melanoma.

lncRNAs refer to a subclass of RNAs with longer than 200 nucleotides that lack protein-coding ability [19]. Although lncRNAs have no protein-coding capacity, they still own some functions, such as transcriptional regulation, mRNA processing, and mRNA post-transcriptional control [20]. Recent research has shown that SLNCR regulates the binding of androgen receptors and EGR1-bound genes in melanoma [21]. However, research on the FRLs in melanoma is lacking, and many FRLs have not been identified. The advances in high-throughput sequencing technology can contribute to finding FRLs biomarkers [22, 23].

In this paper, we aimed to recognize FRLs in melanoma, which may not only provide significant insights into the signaling pathways and molecular mechanism of ferroptosis in melanoma but may also predict its prognosis. Furthermore, we investigated the correlation between ferroptosis and TME in melanoma, guiding the treatment of "cold" melanoma.

## Materials and methods

### Data acquisition

The RNA-sequence transcriptome and clinical data of SKCM were extracted from the TCGA database. The transcriptome data were normalized in FPKM. We obtained the ferroptosis-related genes from FerrDb [24] which provided an updated and comprehensive database of ferroptosis-related regulators, markers, and diseases. According to the human lncRNAs annotation file of GRCh38 from the GENCODE database (http://www.gencodegenes.org/), we acquired the expression data of 4,249 lncRNAs in the TCGA dataset. Then the Pearson correlation coefficients were calculated to define the correlation between the expression of ferroptosis-related genes and corresponding lncRNAs. The FRLs were identified according to the standard the p-value was less than 0.001 (p < 0.001) and the absolute value of the Pearson correlation coefficient was more than 0.3 (|R|> 0.3).

Pearson correlation.

### Identifying prognostic ferroptosis-related lncRNAs and consensus clustering analysis

Univariate and multivariate Cox regression analyses were performed to screen prognostic ferroptosis-related lncRNAs (p < 0.05). To explore the biological characteristics of FRLs in SKCM, we performed an unsupervised cluster analysis on the samples using the “ConsensusClusterPlus” package of R [25]. All tumor samples corresponding to candidate ferroptosis-related lncRNAs were classified into two subgroups: Cluster 1 and Cluster 2. A “survival” package of R was used to explore the survival difference between the two subgroups. Then, the relationship between FRLs and clinicopathological features was shown in heatmaps with the "pheatmap" package. Furthermore, differential expressions of essential genes: melanoma drivers and immune checkpoint genes between two subgroups were identified by the "limma" package. The correlation between the essential genes and prognostic FRLs was detected by the "corrplot" package.

### Comparison of immune-stromal TME components of cluster subgroups

The ESTIMATE algorithm was utilized to quantify the immune and stromal cells in TME and calculated the immune, stromal, and ESTIMATE scores. These scores were analyzed separately to compare the levels of stromal/immune cells in two cluster subgroups.

### Construction and validation of prognostic FRLs

The FRLs were analyzed by the LASSO regression using the "glmnet" package. The samples were randomly allocated into the training group (n = 229) and testing group (n = 227) using the package "caret" in R. The prognostic FRLs were consequently constructed by selecting the optimal penalty parameter λ associated with a minimum tenfold cross-validation. The risk score was calculated through the formula:

Risk score = (coefficient lncRNA1 × expression lncRNA1) + (coefficient lncRNA2 × expression lncRNA2) + … + (coefficient lncRNAn × expression lncRNAn).

The difference in OS between high- and low-risk groups was assessed by the Ka-M analysis using the "survival" package. We compared the sensitivity and specificity differences between the FRLs signatures and other clinicopathological features using “timeROC” and “ggDCA” packages in R [26]. The relationship between FRLs and clinicopathological manifestations was assessed using "ggpubr," "limma," and "pheatmap" packages in R. p < 0.05 was considered statistically significant. All validations were conducted simultaneously in the training and testing cohorts.

### Immunity analysis and gene expression

GSEA software was used to explore the potential cellular pathways and biological processes in the TCGA of SKCM. At the same time, the CIBERSORT [27, 28], CIBERSORT-ABS [29], QUANTISEQ [30, 31], XCELL [32], EPIC [33], ssGSEA [34], MCPCOUNTER [35], and TIMER [36] algorithms were applied to compare differences in cellular components and cellular immune responses between low-risk and high-risk groups based on FRLs signature. The differences in immune responses were compared and displayed in a heatmap. Furthermore, ssGSEA was performed to evaluate the tumor-infiltrating immune cells (TIICs) components in their immune function and 2 subgroups. According to the NCCN guidelines, the association between the gene and risk was assessed using “limma” and “ggupbr” packages in R. Finally, we conducted the Spearman rank correlation test to evaluate the association between risk score and immune cells using “ggExtra” and “limma” packages in R.

### Validation

A study of single cell malignant melanoma transcriptomes defined two main transcriptional states of melanoma cells: the MITF and AXL gene programs.28 We choose the A2058 (MITF) and A375 (AXL) cell lines for our study. Human melanoma cell lines (A2058, A375) and human epithelial cell line HaCaT were purchased from the Shanghai Zhong Qiao Xin Zhou Biotechnology Co., Ltd. These three cell lines were cultured in DMEM (high-glucose) medium (Gibco) containing 10% FBS (Gibco) at 37 °C with 5% CO2 in an incubator. The total RNA of HaCaT, A2058, and A375 cells was extracted using an RNA-easy kit (Vazyme). The primers used in this study were synthesized by GENECREATE (WUHAN GENECREATE BIOLOGICAL ENGINEERING, LTD). Following, the reverse transcription was conducted with the HiFiScript cDNA synthesis kit (Vazyme) to generate cDNA. The qPCR was performed using a LineGene 9600 Plus instrument (Bioer Technology) and 2 × SYBR Green qPCR MasterMix (SEVEN BIOTECH). The CT values were normalized to the expression of the endogenous housekeeping gene GAPDH, and the 2(− ΔΔCt) values were calculated for relative quantification. The reactions were performed in triplicate. The comparisons among multiple groups were conducted by one-way ANOVA. Statistical analyses were carried out using GraphPad Prism 9.0.0 software. All values were normalized relative to GAPDH expression. Primer sets used for qPCR are GAPDH (F: CCAGGTGGTCTCCTCTGA, R: GCTGTAGCCAAATCGTTGT); LINC00861 (F: TGCTCTACTCCTTGGCTAT, R: ACTACGGTAACTCCTATTGC); LINC01094 (F: TGTAAAACGACGGCCAGT, R: CAGGAAACAGCTATGACC).

## Results

### Consensus clustering

**Fig. 1:**
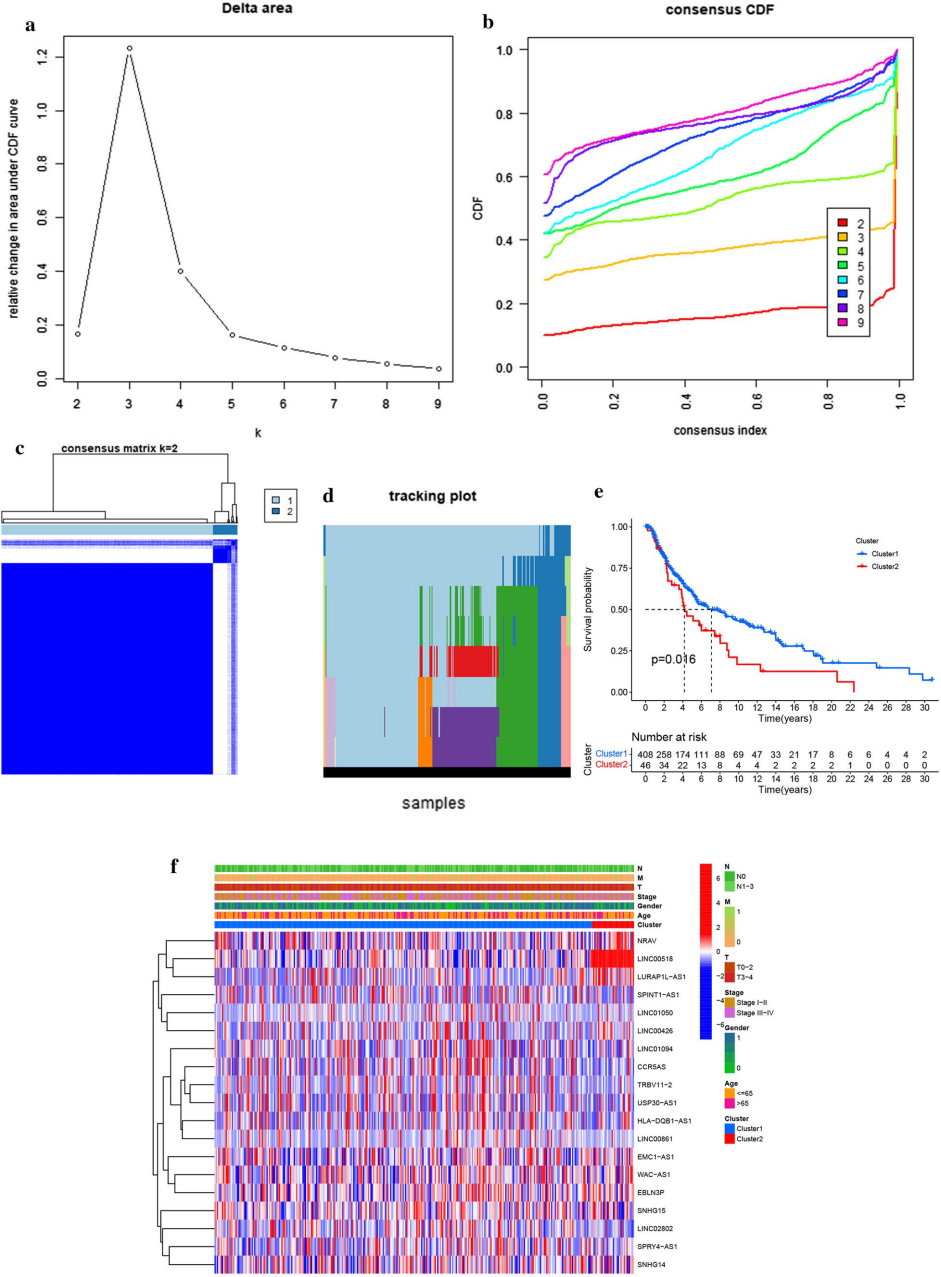
Consistent cluster analysis of SKCM. **a** The consistent cluster CDF when k ranges from 2 to 9. **b** The change in the area under the CDF curve when k is between 2 to 9. **c** The overlap of consistent clustering matrix in clusters for k = 2. **d** The distribution of each sample in the range of k from 2 to 9. **e** Significant difference in K-M survival curves between cluster 1 and cluster 2 (*p* < 0.05). **f** The clinicopathological differences between cluster 1 and cluster 2

When the clustering index “k” ranged from 2 to 9, the optimal value of K was determined to be 2 to obtain the most significant differences between clusters (Fig. 1a, b). Meanwhile, the interference between clusters was minimal when k = 2 (Fig. 1c, d). Subsequently, the SKCM cohort was divided into two clusters: Cluster 1 and Cluster 2. Kaplan–Meier survival analysis found that Cluster 1 had better overall survival than Cluster 2 (Fig. 1e, p < 0.05). The association between the two clusters and clinicopathological characteristics was shown in the heatmap (Fig. 1f).

### Comparison of the clusters

**Fig. 2:**
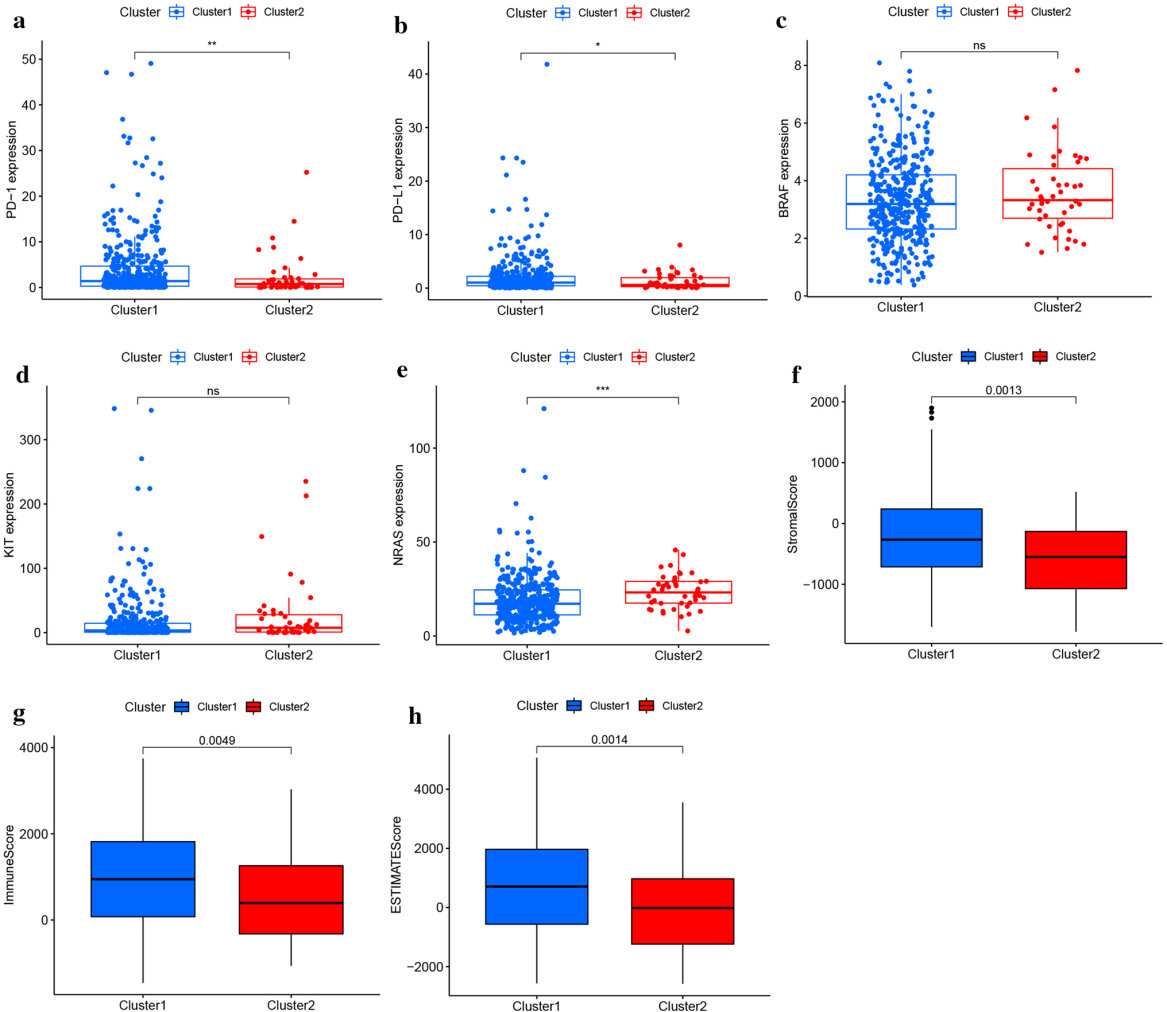
Gene mutations and the tumor microenvironment in the two clusters. **a**–**b** The comparison of immune checkpoint genes expression in the two clusters. **c**–**e** The comparison of commonly mutated gene expression in the two clusters. **f**–**h** Differences in the immune microenvironment scores between Cluster 1 and Cluster 2

The expression of three frequently mutated genes and 2 common immune checkpoint genes (ICGs) in two clusters of SKCM was analyzed. The two ICGs of PD-1 (Fig. 2a, p < 0.01) and PD-L1 (Fig. 2b, p < 0.05) were highly expressed in Cluster 1. In the three commonly mutated genes except for BRAF (Fig. 2c) and KIT (Fig. 2d) genes, the NRAS gene (Fig. 2e, p < 0.001) is highly expressed in Cluster 2. Using ESTIMATE, we found that Cluster 1 had higher stromal (Fig. 2f, p < 0.01), immune (Fig. 2g, p < 0.01), and ESTIMATE (Fig. 2h, p < 0.01) scores than Cluster 2.

### Development of FRLs prognostic signature

**Fig. 3:**
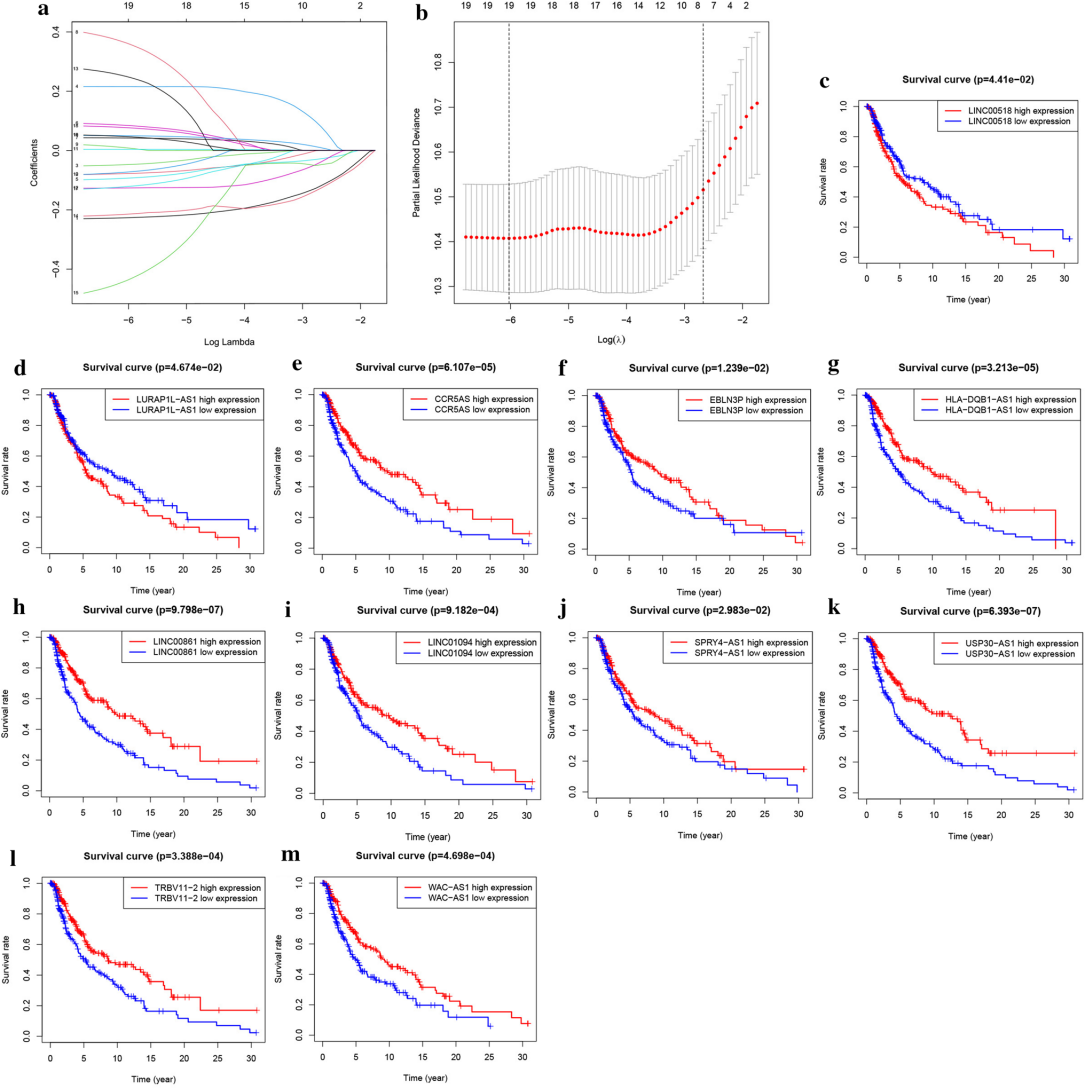
Construction and validation of the FRLs signature. **a**, **b** The LASSO regression was conducted with the minimum criteria. **c**–**m** The Kaplan–Meier survival curves for FRLs in signature

The FRLs signature was established based on the training group. The univariate Cox analysis initially screened 83 significant FRLs, which were further analyzed with LASSO regression, resulting in the final selection of 19 FLRs as independent prognostic factors of SKCM (Fig. 3a, b). The formula of a risk score for each SKCM patient:

Risk score = (SPINT1-AS1) × 0.2153—(SPRY4-AS1) × 0.2267—(WAC-AS1) × 0.0777—(HLA-DQB1-AS1) × 0.0475—(EBLN3P) × 0.0933 + (LINC02802) × 0.0847 + (LINC01050) × 0.0422 + (LINC01094) × 0.3623 + (TRBV11-2) × 0.0090—(EMC1-AS1) × 0.0727 + (LINC00518) × 0.0041—(SNHG14) × 0.1262 + (LINC00861) × 0.2494—(USP30-AS1) × 0.2148—0(CCR5AS) × 0.4381 + (SNHG15) × 0.0499—(LINC00426) × 0.1216 + (NRAV) × 0.0770 + (LURAP1L-AS1) × 0.0468.

The prognostic significance of these 19 FRLs in SKCM was confirmed by K-M analysis. The high expression levels of LINC02802 and LURAP1L-AS1 (Fig. 3c, d) were associated with low survival rates, whereas high expression levels of CCR5AS, EBLN3P, HLA-DQB1-AS1, LINC00861, LINC01094, SPRY4-AS1, USP30-AS1, TRBV11-2, and WAC-AS1 were associated with high survival rates (Fig. 3e–m).

### Validation of the prognostic FRLs signature

**Fig. 4:**
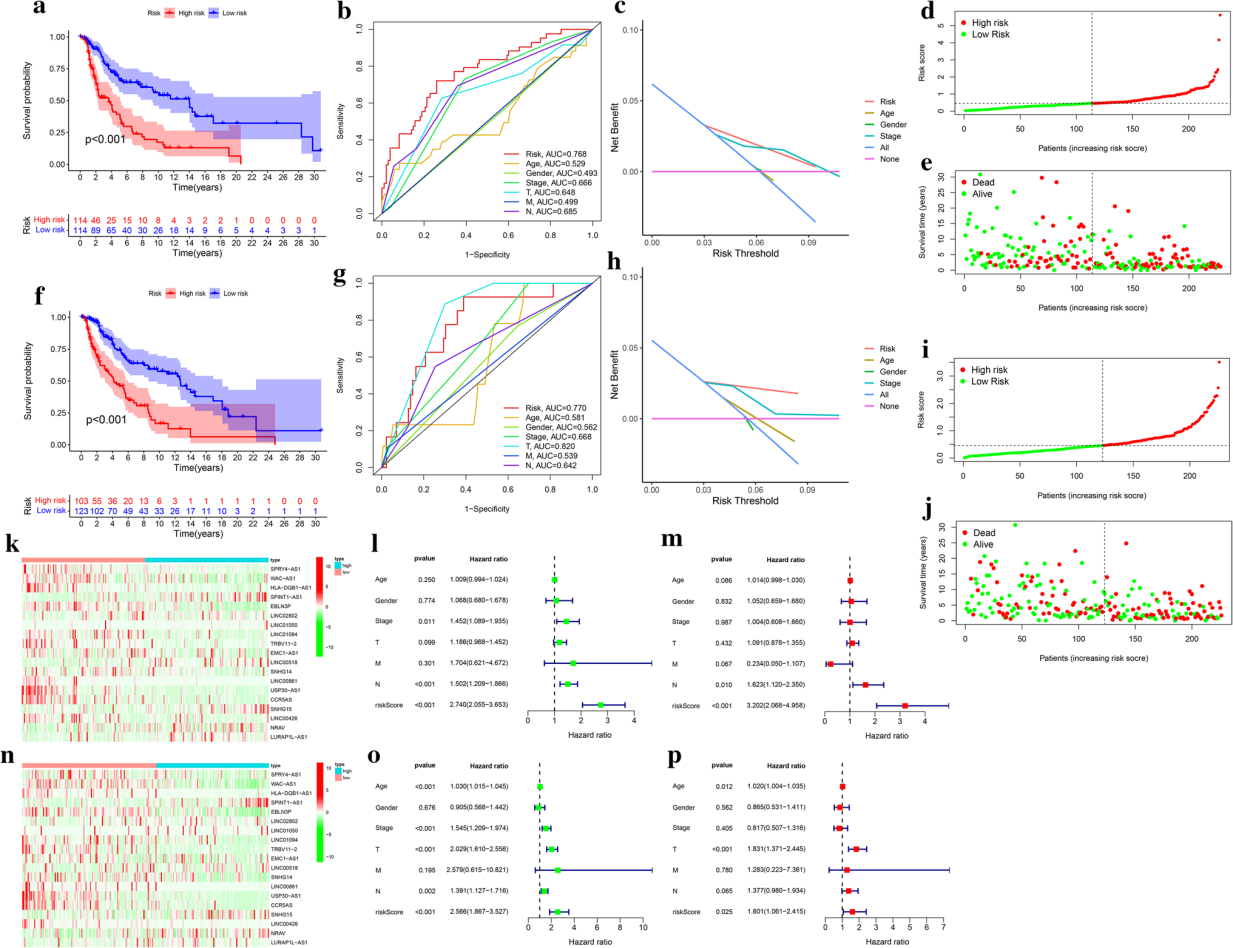
Assessment of the prognostic prediction ability of the FRLs signature. **a**, **f** The Kaplan–Meier survival analysis for high- and low-risk groups in training and testing groups. **b**, **g** The ROC curve was used to evaluate the predictive efficiency of the prognostic signature. **c**, **h** The DCA curve demonstrated that the risk score performed better than traditional clinicopathological factors in predicting the prognosis of SKCM. **d**, **e**, **i**, **j** The distribution plots of the risk score and survival status in the training and testing groups. **k**, **n** The heat map of FRLs for the low-and high-risk groups in the training and testing groups. **l**, **o** The univariate Cox regression of prognostic factors in the training and testing groups. **m**, **p** The multivariate Cox regression of prognostic factors in the training and testing groups

KM survival curve revealed that the high-risk group had a worse survival rate than the low-risk group in the training group (Fig. 4a). Meanwhile, time-dependent ROC and DCA curves were used to assess the FRLs signature. The area under the ROC curve of FRLs signature was 0.768 in the training group. ROC and DCA curves demonstrated that this signature performed better than other clinicopathological factors (Fig. 4b, c). The survival status distribution patterns and risk score indicated that risk score was positively correlated to the mortality of SKCM (Fig. 4d, e). Interestingly, the heatmap exhibited that most FRLs were negatively associated with the risk signature and needed further in-depth studies (Fig. 4f). In univariate Cox regression analysis, there were statistically significant differences among stage, N stage, and risk score (Fig. 4g). In multivariate Cox regression analysis, N stage and risk score were independent prognostic features (Fig. 4h).

To validate the prognostic FRLs signature, the same analyses were conducted in the testing group. The patients in the low-risk group also had better survival status than those in the high-risk group in the testing group (Fig. 4i, l, m). The heatmap, ROC curve, DCA curve, and forest plot of the testing group showed similar results compared to the training group (Fig. 4j, k, n–p). The AUC value was 0.770 in the testing group. All the above results demonstrated that our FRLs signature could stably and accurately predict the prognosis of SKCM.

### Pathway enrichment analyses

**Fig. 5:**
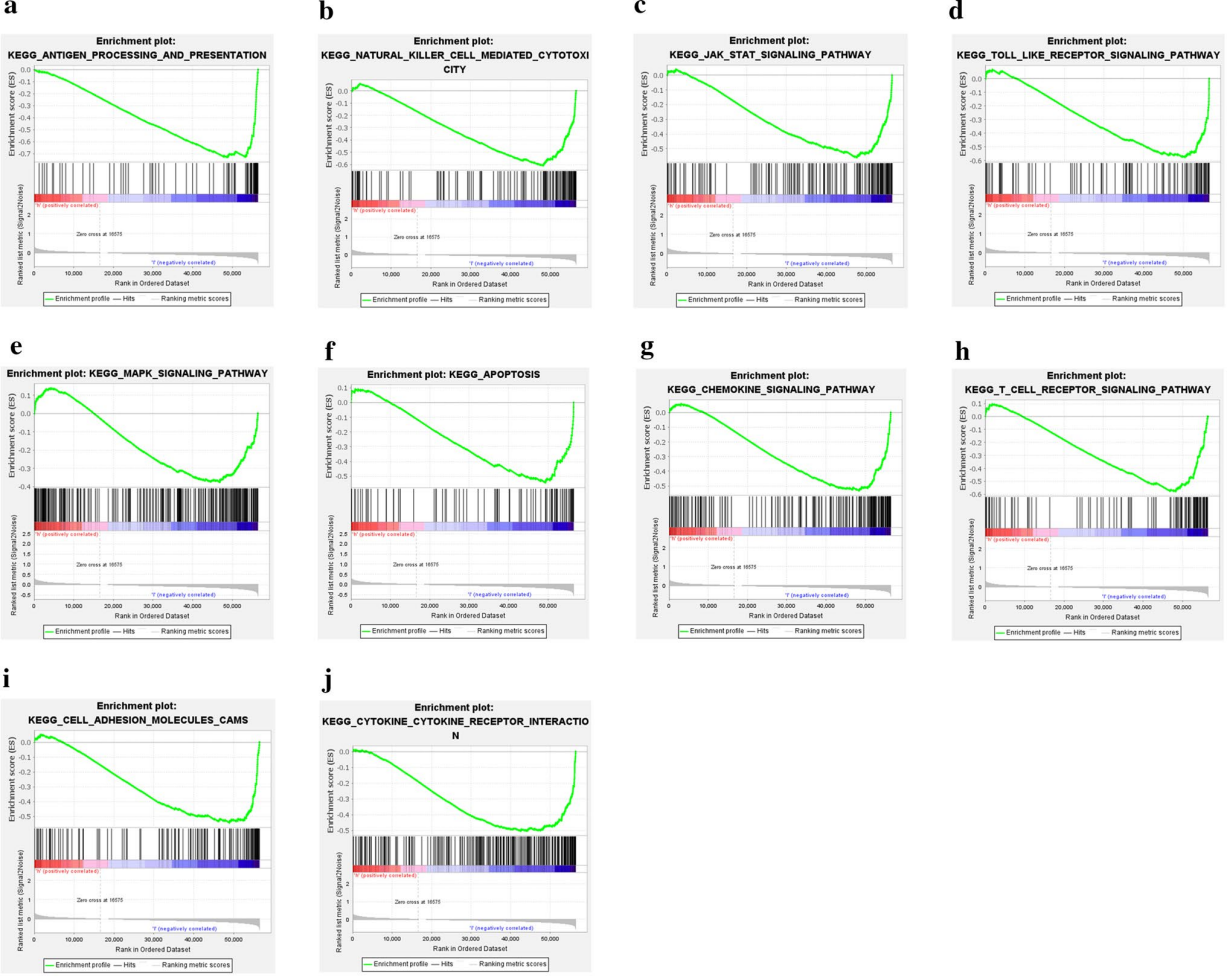
Pathway enrichment analyses. Immune-related signaling pathways and processes were enriched in the low-risk group (**a**–**j**). All p < 0.05, FDR < 0.25

The GSEA revealed that some immune and tumor-related pathways were enriched in the low-risk group, such as antigen processing and presentation, natural killer cell-mediated cytotoxicity, JAK-STAT signaling pathway, Toll-like receptor signaling pathway, MAPK signaling pathway, apoptosis, chemokine signaling pathway, T cell receptor signaling pathway, cell adhesion molecules, and cytokine-cytokine receptor interaction (Fig. 5a–j).

### Immunity and gene expression

**Fig. 6:**
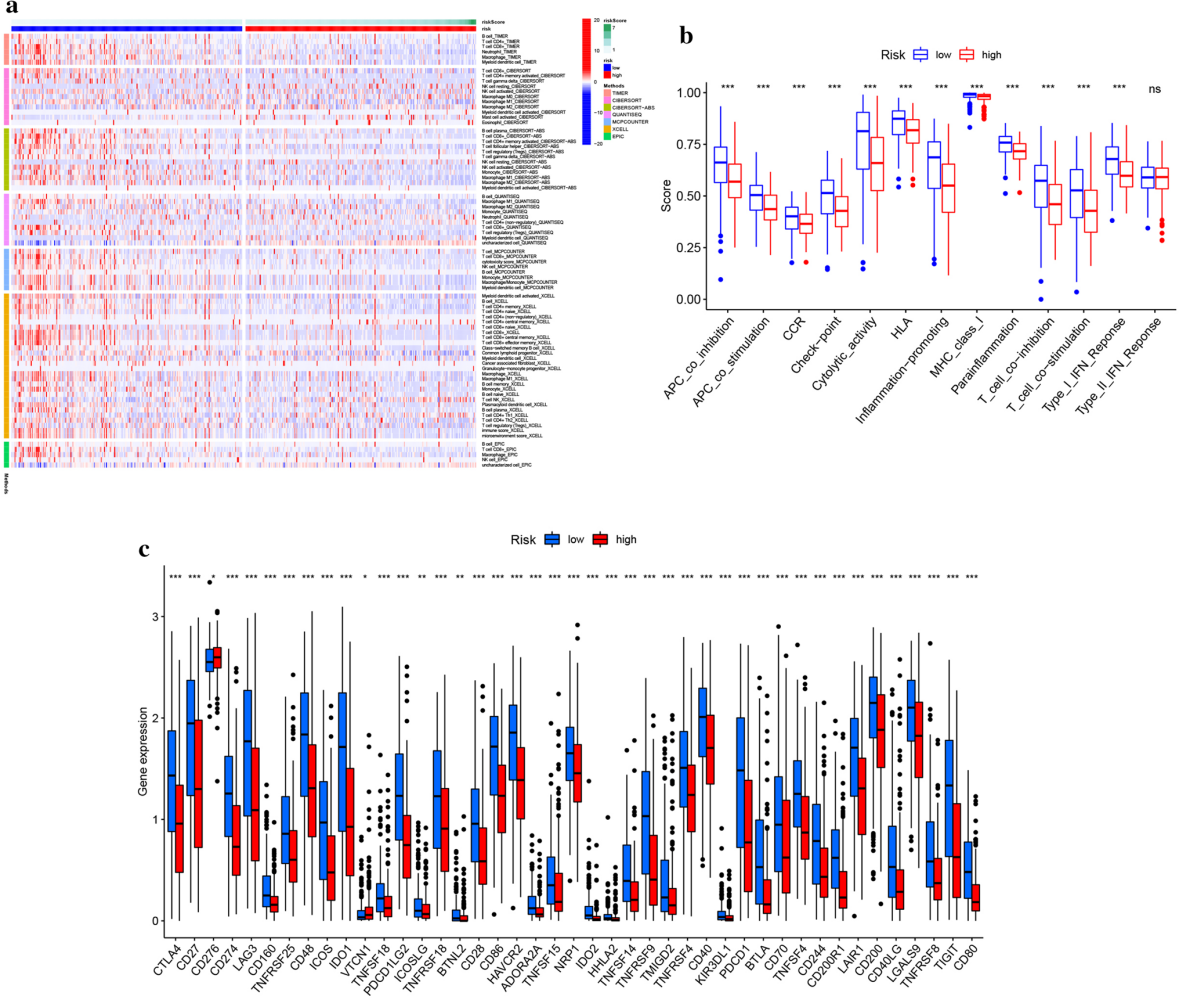
Analysis of differences in immune infiltration and gene expression in two subgroups. **a** Heatmap for immune responses based on TIMER, CIBERSORT, CIBERSORT-ABS, QUANTISEQ, MCPCOUNTER, XCELL, and EPIC algorithms among the high- and low-risk groups in the TCGA cohort. **b** ssGSEA for the association between immune cell subpopulations and related functions. **c** Expression of immune checkpoints among high- and low-risk groups in TCGA cohort

**Fig. 7:**
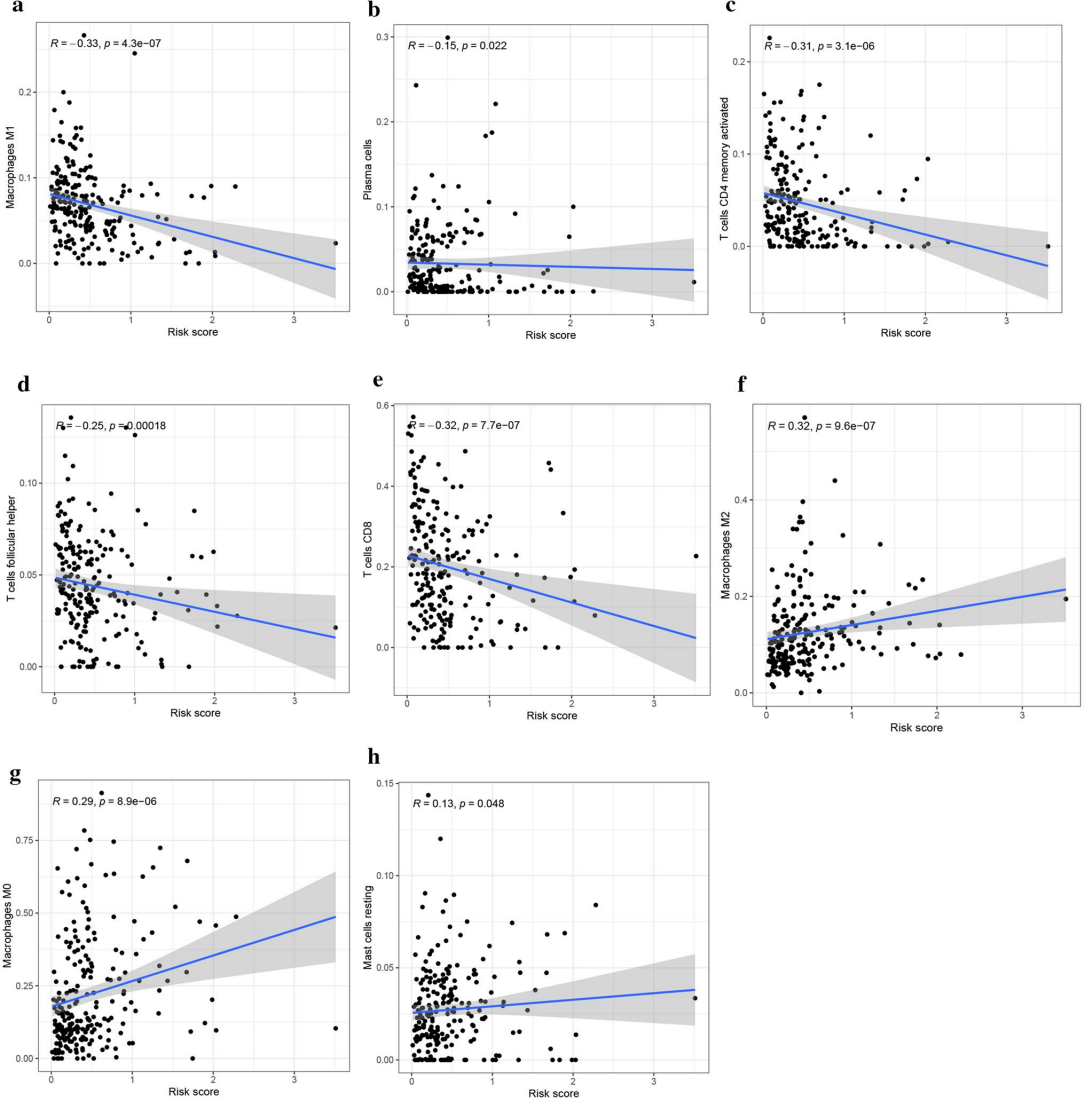
The Relationships between the Risk Score and Tumor-Infiltrating Immune Cells (**a**–**h**)

The heatmap of TIICs based on TIMER, CIBERSORT, CIBERSORT-ABS, QUANTISEQ, MCPCOUNTER, XCELL, and EPIC algorithms between the high- and low-risk subgroups in the TCGA cohort is revealed in Fig. 6a. As shown in Fig. 6b, the ssGSEA showed that APC-co-inhibition, APC-co-stimulation, CCR, checkpoint, cytolytic activity, HLA, inflammation-promoting, MHC class I, parainflammation, T cell co-inhibition, T cell co-stimulation, and type-I-IFN response were significantly different in two groups (p < 0.001). Furthermore, we investigated the difference in the expression of immune checkpoints in the whole TCGA cohort. We found that the low-risk group's expression level of ICGs was higher than that of the high-risk group (Fig. 6c). Finally, Macrophages M1, plasma cells, T cells CD4 memory activated, T cells CD8, T cells follicular helper were found to have negative correlations with the risk score, while macrophages M0, mast cells resting, macrophages M2 had positive correlations with the risk score (Fig. 7, p < 0.001).

### The verification of FRLs expression by qPCR

**Fig. 8:**
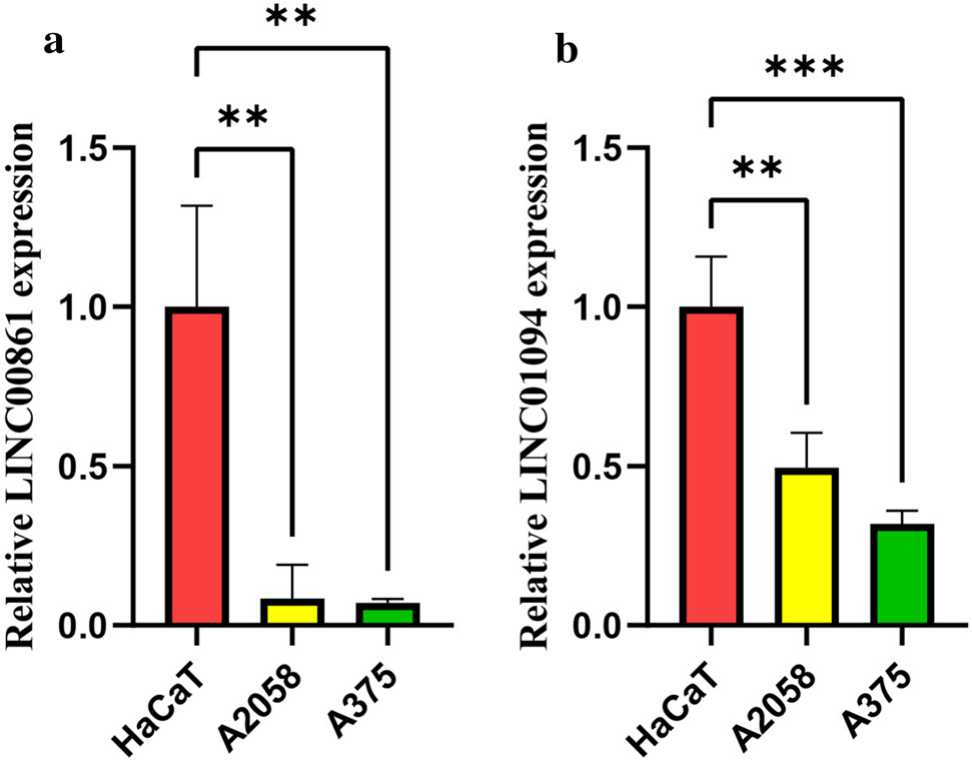
The differential expression of FRLs was detected by qPCR. **a**, **b** Compared with the HaCaT cell line, the FRLs of LINC00861 and LINC01094 were significantly lower in the A2058 and A375 cell lines. *p < 0.05, **p < 0.01, ***p < 0.001, ****p < 0.0001

To further verify the results of FRLs expression between the high- and low-risk groups, qPCR was used to detect these FRLs at the mRNA level in vitro. These results of qPCR confirmed that the expression of LINC00861 and LINC01094 in A2058 and A375 cell lines (SKCM tumor cells) was significantly lower than that in the HaCaT cell line (normal skin cells) (Fig. 8a,b). This finding was consistent with the differential expression of FRLs genes between low- and high-risk groups, further validating the accuracy and reliability of this signature model.

## Discussion

The induction of programmed cell death is considered to be the most promising approach in cancer therapy and ferroptosis has recently been researched in tumor therapies. Ferroptosis-related biomarkers have been demonstrated to be robust predictors of cancer prognosis and antitumor efficacy [37–40]. However, previous studies about ferroptosis-related genes focused only on protein-coding genes. Most studies had grossly neglected the essential role of lncRNAs in tumor initiation, progression, and metastasis. In fact, many lncRNAs participated in tumorigenesis and cancer progression, and thus they could serve as novel biomarkers for tumor diagnosis and treatment [41–43]. Many lncRNAs induced ferroptosis in cancer cells and tumor immunity [44–46]. Thus, they could be promising tumor markers with clinical significance. With increasing evidence suggesting the role of lncRNAs in ferroptosis through epigenetic regulation, we considered that a comprehensive assessment of the prognosis of FRLs in SKCM was necessary.

In this paper, we established a 19 FRLs prognostic signature in the training group by univariate Cox and LASSO regression analyses. According to the FRLs signature, the risk score was calculated and used to predict SKCM patients' survival. The survival rate of SKCM patients with low-risk scores was better than that of those with high-risk scores. The ROC and DCA curves analyses further demonstrated that the FRLs signature could accurately predict the survival of patients with SKCM. Furthermore, this signature also performed very well in the testing group. Therefore, we speculated that the 19 FRLs signature was significantly related to the progression and prognosis of SKCM and could be a powerful indicator of the clinical outcome in SKCM patients. Here, we studied the correlation between FRLs and SKCM prognosis. The SKCM predictive signature was successfully constructed with 19 FRLs, including 13 protective factors (CCR5AS, EBLN3P, HLA-DQB1-AS1, LINC00861, LINC01094, SPRY4-AS1, USP30-AS1, TRBV11-2, and WAC-AS1) and six risk factors (LINC00518 and LURAP1L-AS1). For example, LINC00518 is a tumor-promoting factor in multiple tumors, including lung adenocarcinoma [47], cervical [48], breast [49], and non-small cell lung cancer [50]. Similarly, it was found that LINC00518 promoted the migration, invasion, and metastasis of melanoma through the miR-204-5p/AP1S2 [51] and miR-33a-3p/HIF-1α [52] axes, which is consistent with our finding that LINC00518 serves as a risk factor for SKCM. Although there is currently little direct evidence to verify the correlation of NRAV, EBLN3P, SPINT1-AS1, LINC01094, SNHG14, SNHG15, SPRY4-AS1, USP30-AS1, and TRBV11-2 with SKCM, many studies have suggested that they play significant roles in multiple tumors. NRAV could be used to predict immune checkpoint inhibition and prognosis in hepatocellular carcinoma [53, 54]. EBLN3P has different roles in multiple tumors. For example, EBLN3P regulates DOCK4 expression via sponging miR-144-3p competitively, thereby participating in liver cancer's progression [55]. In addition, overexpression of EBLN3P caused carcinogenesis of colorectal tissues by decreasing the suppressive effects of miR-323a-3p on UHMK1 expression [56]. The SPINT1-AS1 promoted the progression of breast cancer by regulating miR-let-7a/b/i-5p [57]. In contrast, LINC00861 could inhibit cervical cancer's progression by competitively sponging miR‑513b‑5p [58]. The LINC01094 promotes ccRCC development by miR-224-5p/CHSY1 or upregulating SLC2A3 via microRNA-184 [59, 60]. However, only a few studies are exploring the involvement of 19 lncRNAs in the ferroptosis process. Their possible regulatory functions in the process of ferroptosis need further experimental validation.

Afterward, we investigated potential signaling pathways associated with the 19 FRLs and found some ferroptosis-related signal pathways. JAK-STAT signaling pathway, Toll-like receptor signaling pathway, and MAPK signaling pathway were significantly enriched in the low-risk group. Previous papers have demonstrated that Toll-like receptor, MAPK- and JAK-STAT signaling pathways participated in ferroptosis in diverse diseases [61–63]. For example, ferroptosis of osteosarcoma and hepatocellular carcinoma cells was induced by activating MAPK signaling pathway [64, 65]. However, no strong evidence exists on how these signaling pathways participate in the ferroptosis of SKCM. Furthermore, some significant immune-related functions and pathways were also enriched in the low-risk group. Consequently, we could reasonably assume that ferroptosis is strongly associated with antitumor immunity in SKCM. The ferroptotic cells could release signals to recruit antigen-presenting cells to them [66]. CD8 + T cells were discovered to play an antitumor function by inducing tumor ferroptosis [67]. These outcomes indicated that ferroptosis is linked to antitumor immunity, which was consistent with our speculation.

GSEA suggested that immune-related pathways and functions were significantly enriched in the low-risk group. Meanwhile, we found that the low-risk group had a higher level of tumor immune cell infiltration than that of the high-risk group. Furthermore, SKCM in the low-risk group based on the FRLs signature had higher expression levels of ICGs. Therefore, SKCM in the low-risk group was immunologically “hot”, which was more sensitive to the treatment of ICIs, while SKCM in the high-risk group was immunologically “cold”, which was less sensitive to the treatment of ICIs [68]. The results suggested that our FRLs signature could predict the efficacy of ICIs for SKCM.

Undoubtedly, our study had some limitations. First, the FRLs signature was only established and processed from the TCGA database. Although we conducted internal validation and qPCR verification, the signature’s reliability and robustness still need to be further confirmed with real datasets. Second, the association between FRLs and antitumor immunity was preliminarily explored. So, we need to determine detailed mechanisms of action through further experiments. Finally, the detailed and specific FRLs underlying the signature in SKCM are yet unknown. We should strive to investigate their relationship with specific laboratory experiments.

## Conclusions

In summary, our study identified a novel FRLs signature that accurately predicted the prognosis of SKCM. Furthermore, through functional enrichment analysis, we explored the role of these FRLs in the immunity and ferroptosis of SKCM, providing new insights for further understanding of the molecular mechanisms in the development and progression of SKCM. These FRLs may be involved in antitumor immunity and act as therapeutic targets for SKCM.

## Data Availability

The original data of the study can be obtained from the corresponding author on reasonable request.
